# Comprehensive liquid biopsy analysis as a tool for the early detection of minimal residual disease in breast cancer

**DOI:** 10.1038/s41598-022-25400-1

**Published:** 2023-01-23

**Authors:** Dimitra Stergiopoulou, Athina Markou, Areti Strati, Martha Zavridou, Eleni Tzanikou, Sophia Mastoraki, Galatea Kallergi, Vassilis Georgoulias, Evi Lianidou

**Affiliations:** 1 Analysis of Circulating Tumor Cells Lab, Lab of Analytical Chemistry, Department of Chemistry, National and Kapodistrian University of Athens, 15771 Athens, Greece; 2 Division of Genetics, Cell and Developmental Biology, Department of Biology, University of Patras, 26500 Patras, Greece; 3 First Department of Medical Oncology, METROPOLITAN General Hospital, 264, Mesogion Av, Cholargos, Athens, Greece

**Keywords:** Breast cancer, Breast cancer

## Abstract

Liquid biopsy (LB) provides a unique minimally invasive tool to follow-up cancer patients over time, to detect minimal residual disease (MRD), to study metastasis-biology and mechanisms of therapy-resistance. Molecular characterization of CTCs offers additionally the potential to understand resistance to therapy and implement individualized targeted treatments which can be modified during the disease evolution and follow-up period of a patient. In this study, we present a long-term follow-up of operable breast cancer patients based on a comprehensive liquid biopsy analysis. We performed a comprehensive liquid biopsy analysis in peripheral blood of 13 patients with early-stage operable breast cancer at several time points for a period of ten years, consisting of: (a) CTC enumeration using the CellSearch system, (b) phenotypic analysis of CTCs using Immunofluorescence, (c) gene expression analysis, in EpCAM^(+)^ CTCs for *CK-19, CD24,CD44, ALDH1,* and *TWIST1*, (d) analysis of *PIK3CA* and *ESR1* mutations in EpCAM^(+)^ CTCs and corresponding plasma ctDNA and (e) DNA methylation of *ESR1* in CTCs. 10/13 (77%) patients were found negative for LB markers in PB during the whole follow-up period, and these patients did not relapse during the follow-up. However, 3/13(18%) patients that were positive for at least one LB marker relapsed within the follow-up period. The molecular characteristics of CTCs were highly different even for the same patient at different time points, and always increased before the clinical relapse. Our results indicate that liquid biopsy can reveal the presence of MRD at least 4 years before the appearance of clinically detectable metastatic disease demonstrating that a comprehensive liquid biopsy analysis provides highly important information for the therapeutic management of breast cancer patients.

## Introduction

Minimal residual disease (MRD) detection and monitoring remains a high challenge for the management of patients with solid tumours^1,2^. A considerable number of patients with breast cancer will develop metastasis within five years of primary tumor resection despite initially being free of detectable metastases depending on the tumor type and stage. In ER(+) breast cancer, after 5 years of adjuvant endocrine therapy, breast cancer recurrences continue to occur steadily throughout the study period from 5 to 20 years^1^. A significant proportion of these early-stage breast cancer patients that seemingly response to treatment have occult micrometastases or MRD that perseveres after initial therapy as a potential source of subsequent metastatic relapse at distant sites. The early identification of MRD in individual patients is highly challenging; towards this goal, real-time high-sensitivity liquid biopsy (LB) assays are highly promising and offer a great potential to address this^2^.

LB is a minimally invasive approach that is mainly based on the analysis of circulating tumor cells (CTCs) and circulating tumor DNA (ctDNA)^3–5^. LB is an important tool in the fight against cancer, since it provides the ability to monitor in serial samples a molecular portrait of the tumor in real time^3,6,7^. CTC analysis currently presents a powerful tool for the management of advanced- and early-stage cancer patients; especially CTC molecular characterization offers the unique potential to better understand the biology of metastasis and resistance mechanisms to specific treatments^3,6^. In the early stages of cancer, CTCs are usually detected at very low numbers and are characterized by a high heterogeneity^7^. It is more than ten years that CTCs enumeration performed in the CellSearch system has been FDA-cleared as a prognostic marker and has been associated with progression-free survival (PFS) and overall survival (OS) in early and metastatic breast, metastatic prostate and colorectal cancer^3,8,9^. The STIC CTC trial has shown that CTC count can be a reliable biomarker for choosing between chemotherapy and single-agent endocrine therapy as the first-line treatment in hormone receptor–positive HER2-negative metastatic breast cancer^10^. Müller et al.^11^ demonstrated that the presence of even one CTC with strong HER2 staining was associated with shorter OS, supporting a biological role of HER2 expression on CTCs.

During the last years CTC biology is under intense study^12^. Beyond CTC enumeration, molecular characterization of CTCs at the gene expression level has the potential to clarify the critical signalling pathways involved in metastasis biology and even improve patient management^3,6^. We have previously shown that the detection of cytokeratin-19 (*CK-19*) transcripts in CTCs by RT-qPCR in breast cancer patients is of prognostic significance before, during and after adjuvant chemotherapy^13–20^. It is also known that the expression of epithelial-mesenchymal transition (EMT) and stem cell markers like vimentin, N-cadherin, SNAI1 (also known as Snail), SNAI2 (also known as Slug), TWIST, zinc finger E-box binding homeobox 1 (ZEB1), and ZEB2 in CTCs is associated with an invasive phenotype^21^. Moreover, a subset of tumor cells with stem cell properties can contribute to tumor initiation and growth but can also play an important role to the development of metastasis and drug resistance^3^. Cancer stem cell (CSC) markers like *ALDH1, CD24, CD44,* and *CD133* have been detected in CTCs^22–27^, and their clinical relevance has already been reported^15,28^.

In breast cancer, detection of *PIK3CA* mutations is highly important, since recently there are specific targeted therapies developed^29^. Detection of *PIK3CA* in CTCs could have important clinical applications for the follow-up of these patients. However, this is very challenging, since CTCs are heterogeneous and cells carrying these mutations consist often a minority in the CTC population. Our group has developed highly sensitive and specific assays for the detection of *PIK3CA* hotspot mutations (E545K, H1047R)^30–32^ and *ESR1* mutations^33^ in EpCAM^(+)^ CTC of early and metastatic breast cancer patients. There are also several studies in which the *PIK3CA* mutational status of CTCs has been investigated at the single cell level revealing the heterogeneity of CTC in the same patient^34–36^. *ESR1* gene mutations consist one of the resistant mechanisms to endocrine therapies for ER-positive metastatic breast cancer patients^37^ and have been also detected in single CTCs exposing important information about their mutational heterogeneity and subsequently for the resistance to therapies^38,39^. Beyond mutations, epigenetic modifications, especially DNA methylation of tumor and suppressor genes’ promoters, play an important role in cancer development^40^. We have recently reported that methylation of the *ESR1* promoter is an alternative and additional mechanism of *ESR1* inactivation leading to resistance to hormone treatment in breast cancer^41^.

In this study, we present for the first time a comprehensive liquid biopsy analysis for 13 patients all diagnosed with early breast cancer, based on a long-term follow-up. Our analysis was based on CTC enumeration, CTC phenotypic characterization and molecular monitoring of CTCs at the gene expression, DNA mutation and DNA methylation level, and on corresponding plasma cell free DNA for DNA mutation and DNA methylation level. Our results indicate that a comprehensive liquid biopsy analysis provides valuable and important information for the therapeutic management of breast cancer patients since it is the only way to shed light into the black box that was sealed till now between the information taken from the primary tumor and from a distant metastatic site. Our results demonstrate that liquid biopsy analysis could reveal the presence of MRD years before the appearance of clinically detectable metastatic disease and progression of disease.

## Results

### *CK-19* mRNA expression

**Figure 1:**
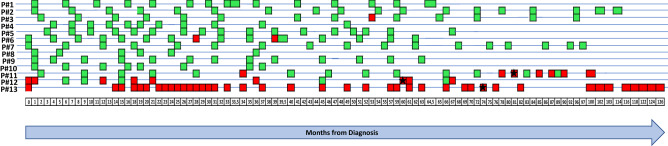
Molecular analysis of EpCAM^(+)^ CTC fractions for *CK-19* mRNA expression, DNA mutations (*PIK3CA*, *ESR1*) and CTC enumeration. Stars represent the time of relapse/metastasis (red: positive, green: negative).

The presence of *CK-19* transcripts was assessed in all patient samples at different time points during the follow-up period (Fig. 1). Among the ten patients remained free from disease progression over the ten years of follow-up, *CK-19* transcripts were not detected in eight (8/10, 80%) of them (Pt#1, Pt#2, Pt#4, Pt#5, Pt#7, Pt#8, Pt#9, Pt#10) at all time points. The other two patients (Pt#3, Pt#6) were found positive for *CK-19* transcripts; more specifically, Pt#3 was found positive for *CK-19* transcripts on month 53 after diagnosis and Pt#6 on months 28 and 39, while *CK-19* transcripts were not detected at all previous and ensuing time points.

All breast cancer patients (Pt#11, Pt#12, Pt#13) that were positive for *CK-19* transcripts early on, and at various time points later developed metastasis (Fig. 1). Pt#11 at the initial time point was found negative for *CK-19* transcripts. However, after 78 and 81 months after initial diagnosis, high levels of *CK-19* transcripts were detected in CTCs and two months later progression of disease (PD) was confirmed by detecting metastatic lesions on adrenal gland and liver. During the metastatic phase of the disease, all samples analyzed for *CK-19* transcripts were found positive and PD was confirmed a few months later at each time point. This patient died three years after metastasis was documented clinically and by imaging studies and one month after a new progression. Pt#12 was found positive for *CK-19* transcripts 61 months since diagnosis and one month later after confirmation of PD (Fig. 1). In Pt#13, no *CK-19* transcripts were detected before the initiation of adjuvant chemotherapy. On September 2014, a significant increase in the number of *CK-19* transcripts was observed. Eight months later a liver metastasis was confirmed by a liver biopsy. During the metastatic phase of the disease (Fig. 1) all samples analyzed for *CK-19* transcripts were found positive.

### Detection of *ESR1* & *PIK3CA* mutations

**Figure 2:**
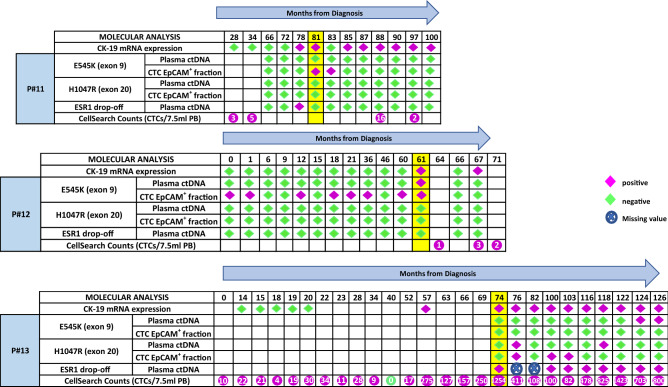
Molecular analysis of EpCAM^(+)^ CTC fractions for *CK-19* mRNA expression, DNA mutations (*PIK3CA*, *ESR1*), CTC enumeration and corresponding plasma ctDNA for P#11, P#12 and P#13 (yellow: time of relapse/metastasis, purple: positive, green: negative).

We further analyzed all available plasma-cfDNA and CTC-derived gDNA samples from the three breast cancer patients (Pt#11, Pt#12, Pt#13) that developed metastasis at different time points for *ESR1* and *PIK3CA* mutations (Fig. 2). We first used a drop-off ddPCR to detect *ESR1* mutations in plasma-ctDNA samples at different time points^41^. Pt#12 was found negative for *ESR1* mutations in plasma-ctDNA at all the time points tested; this patient experienced a clinical objective response to endocrine treatment and remained free from disease progression at the time of analysis for 61 months since diagnosis. Pt#13 was found positive for *ESR1* mutations in plasma-ctDNA consistently at five different time points during the follow-up period (Fig. 2). This patient later developed resistance to endocrine therapy. In parallel, all available plasma-cfDNA and CTC-derived gDNA samples were analyzed for *PIK3CA* mutations. *PIK3CA* E545K mutation was detected in 2/10 gDNA samples from Pt#11 extracted from EpCAM^(+)^ fractions (Fig. 2). Pt#12 was found positive for *PIK3CA* E545K mutation in 8/14 gDNA samples extracted from EpCAM^(+)^ fractions and in 1/14 paired plasma-ctDNA samples (Fig. 2). Pt#13 was found positive for *PIK3CA* H1047R mutation in 3/10 gDNA samples extracted from EpCAM^(+)^ cell fractions and in 2/10 paired plasma-ctDNA samples. *PIK3CA* E545K mutation was detected in 2/10 paired plasma-ctDNA samples.

### Comprehensive liquid biopsy analysis for Pt#13

**Figure 3:**
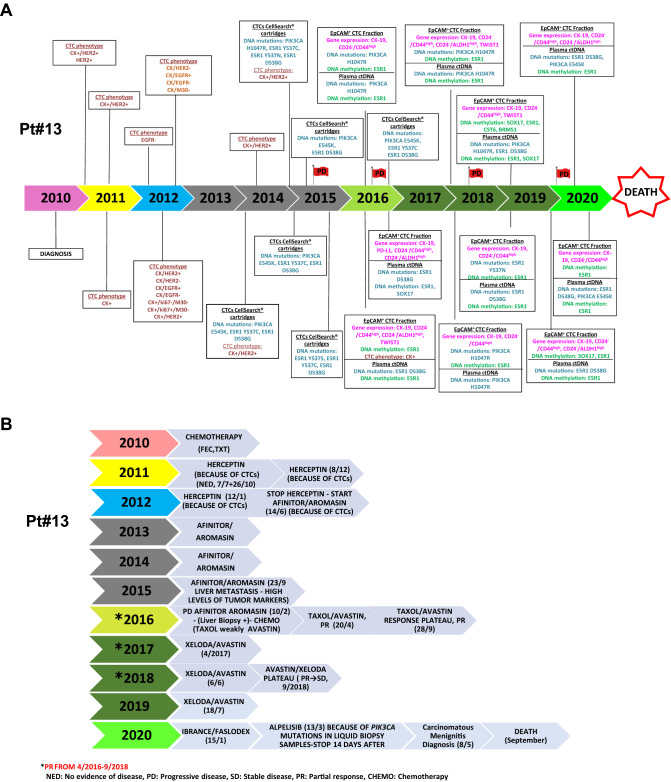
P#13: (**A**) timeline of disease course from diagnosis onward including info on the various treatments, and the phenotypic characterization of CTCs based on IF. The red flags represent onset of relapses. (**B**) Timeline of treatments.

An overview presenting timeline of the disease course from diagnosis onward, therapeutic interventions and the results for CTC enumeration, and the phenotypic and molecular characterization of CTCs at different time points for Pt#13 is shown in Fig. 3A; therapeutic interventions for this patient at different time points are shown in Fig. 3B.

- CTC enumeration and therapeutic interventions

**Figure 4:**
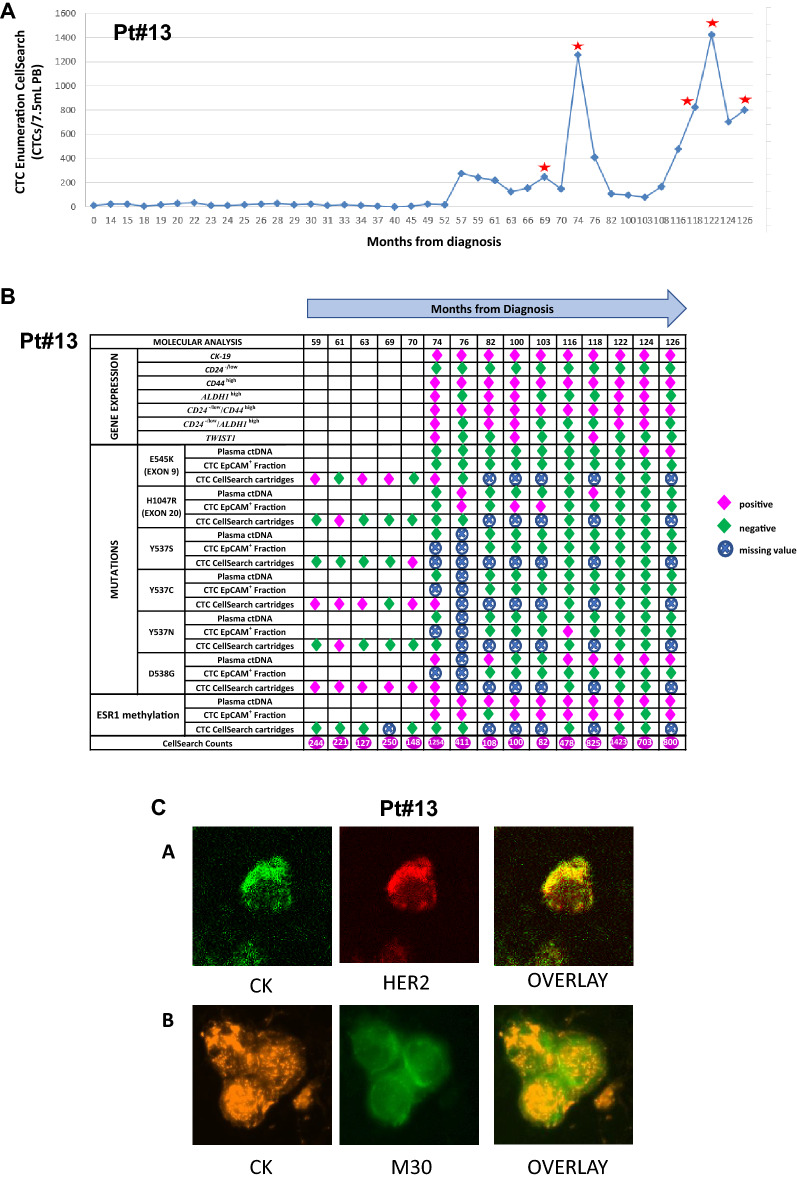
P#13: (**A**) CTC enumeration values (CTCs/7.5 mL PB) based on CellSearch, during the timeline of the disease (red stars represent the time of progression disease). (**B**) Molecular analysis of EpCAM^(+)^ CTC fractions and CTCs from CellSearch cartridges for gene expression, *PIK3CA* and *ESR1* mutations and *ESR1* methylation, and corresponding plasma ctDNA (purple: positive, green: negative, crossed circle: missing value). (**C**) Representative images of CTCs by immunofluorescence experiments and confocal laser scanning microscopy analysis.

CTC enumeration using the CellSearch system was performed at regular time points during the follow-up period (Fig. 4A). Before the initiation of adjuvant systemic chemotherapy, CTC enumeration revealed the presence of 10CTCs/23 mL PB (time point: 0 months, Fig. 4A). The patient received a dose-dense chemotherapy (AC every 2 weeks followed by 4 cycles docetaxel every 2 weeks followed by adjuvant radiotherapy^42,43^. At the time of radiotherapy completion (Fig. 3B), a new CTC enumeration test revealed the presence of 7CTCs/23 mL PB. During adjuvant hormone treatment (LH/LH analogs and tamoxifen) (time point: 15 months, Fig. 4A) a slight increase of the CTC number (10CTCs/23 mL PB) was observed which was further confirmed 3 months later. On January 2011, letrozol was added instead of tamoxifen (Fig. 3B) and a more detailed characterization of CTCs by double immunofluorescence demonstrated that practically all the detected cells were CK^(+)^/HER2^(+)^ and CK^(+)^/Vimentin^(+)^ (Fig. 3A). Based on these results the patient was enrolled in a phase II randomized clinical trial of secondary adjuvant Trastuzumab versus standard hormone treatment^42^ and was allowed to receive trastuzumab along with hormone treatment; treatment (April 2011–December 2011) resulted in a clear decrease of the absolute number of CTCs (time point: 15 months, Fig. 4A). On February 2012 a significant increase of CTCs was noted, (time point: 26 months, Fig. 4A) bearing the HER2^(−)^, EGFR^(+)^, Ki67^(−)^, M30^(−)^ phenotype. Trastuzumab was discontinued and a 3-month treatment with lapatinib was administered based on the EGFR + phenotype of the cells without success (time point: 26 months, Fig. 4A). On July 2012, the patient received afinitor/aromasin with a clear decrease of the CTCs’ number from 25/23 mL PB (time points: 26 months and 37 months, Fig. 4A) until September 2014 when 275 CTCs/23 mL PB reappeared in the blood. A parallel analysis based on IF in the same PB samples (Fig. 3A) has shown that these cells were CK^(+)^/HER2^(−)^ (time point: 57 months, Fig. 4A). At this period tumor markers were moderately increased for the first time (CEA: 6 mg/mL and CA19.9: 51 IU/mL). On December 2014, whole bone scintigraphy demonstrated lesions suspicious for bone metastases which, however, could not be confirmed by MRIs. Until September 2015 the patient was without medical complains and the imaging studies with CT scans every 4 months were practically negative; the patient continued to receive afinitor/aromasin despite the clinical suspicion of disease progression. At this period the MRI of the abdomen as well as a PET scan demonstrated the presence of multiple hypodense lesions of the liver and a liver biopsy has confirmed the metastatic nature of the lesions (ER+, PR+, HER2-neg, Ki67 = 50%), compatible with the primary tumor. The patient received front line chemotherapy with weekly paclitaxel + bevacizumab which resulted in a significant decrease of the CTC number despite the stable disease which was documented as best clinical response 1254 CTCs/7.5 mL PB (time point: 74 months, Fig. 4A) and 108/7.5 mL PB, (time point: 82 months, Fig. 4A), respectively. Subsequently, because of a new increase of CTC count [CTCs: 411/7.5 mL PB (time point: 76 months, Fig. 4A)] a combination of capecitabine/bevacizumab was initiated with major objective response after 3 cycles, resulting to a long lasting clinical partial response with a decreased CTC count [CTCs:411/7.5 mL PB (time point: 76 months, Fig. 4A), to 108 CTCs/7,5 mL PB (time point: 82 months, Fig. 4A), to CTCs: 82 CTCs/7.5 mL PB (time point: 103 months, Fig. 4A)]; however, the CTC counts were increased again (478 CTCs/7.5 mL PB time point: 116 months, Fig. 4A) and were further increased despite under a combination of a CDK4/6 inhibitor/Faslodex [1423CTCs/7.5 mL PB (time point: 122 months, Fig. 4A)]. On March 2020, the number of CTCs was decreased (703 CTCs/7.5 mL PB, time point: 124 months, Fig. 4A) while on May 2020, at the time of carcinomatous meningitis diagnosis, an increase of CTC values was observed.

- Molecular analysis of CTCs and corresponding ctDNA

#### Gene expression

##### *CK-19* mRNA

Before the initiation of adjuvant chemotherapy, CK-19 transcripts were not detected in PB of Pt#13. On September 2014, CK-19 transcripts were observed: this has been confirmed by a parallel analysis in the CellSearch, using the same blood draw (275 CTCs/7.5 mL of blood; time point: 57 months, Fig. 4A). Eight months later (time point: 66 months, Fig. 4A) a liver metastasis, confirmed by a new biopsy, was detected. During the metastatic phase of the disease (February 2016 until May 2020; time points: 74–103 months, 116–126 months, Fig. 4B) all samples analyzed for CK-19 transcripts were found positive.

##### *CD24, CD44, ALDH1, TWIST1* mRNA

The *CD24*^*−/low*^*/CD44*^*high*^ profile was detected in all samples (time points: 74–103 months, 116–126 months, Fig. 4B) and the *CD24*^*−/low*^*/ALDH1*^*high*^ in 5/10 (50%) samples (time points: 74, 82, 100, 122, 124 months, Fig. 4B). *TWIST1* overexpression was detected in 3/10 (30%) samples (time points: 74, 100, 118 months, Fig. 4B). It should be mentioned that TWIST1 overexpression was detected in samples with *CD24*^*−/low*^*/CD44*^*very high*^ profile.

#### DNA mutations

##### *PIK3CA* mutations

*PIK3CA* hotspot mutations were detected in 25 gDNA samples extracted from CellSearch cartridges (n = 15) and EpCAM^(+)^ CTC fractions (n = 10) (Fig. 4B). In particular, *PIK3CA* hotspot mutations were detected in 5/15(36%) gDNA samples extracted from CTCs isolated from CellSearch cartridges. E545K mutation was detected in 4/15 (27%) samples (time points: 59, 63, 69, 74 months, Fig. 4B) and H1047R mutation in another 1/15 (7%) sample (time point: 61 months, Fig. 4B). All samples found positive for *PIK3CA* mutations were collected one year after the patient started to receive Afinitor/Aromasin therapy. In addition, *PIK3CA* H1047R hotspot mutation was detected in 3/10 (30%) gDNA samples extracted from EpCAM^(+)^ CTC fractions (time points: 76, 100, 103 months, Fig. 4B). It is important to mention that *PIK3CA* mutations were not detected in gDNA from the primary tumor (FFPEs) when the same highly sensitive methodology was used. There was no concordance in the findings for the detection of *PIK3CA* mutations between EpCAM^(+)^fractions and CellSearch cartridges. On March 2020 the patient received Alpelisib based on the detection of *PIK3CA* mutations in ctDNA and CTCs for a period of 2 months. But the drug was discontinued after two months due to serious adverse events. In paired plasma-ctDNA samples*, PIK3CA* H1047R hotspot mutation was detected in 2/10 (20%) (time points: 76, 118 months, Fig. 4B) and *PIK3CA* E545K in 2/10 (20%) (time points: 124, 126 months, Fig. 4B). It is noteworthy that the same mutations were also detected in CTCs obtained from both CellSearch cartridges and EpCAM^(+)^ fractions.

##### *ESR1* mutations

*ESR1* hotspot mutations, were detected in 20 gDNA samples extracted from CellSearch cartridges (n = 12) and EpCAM^(+)^ CTC fractions (n = 8) (Fig. 4B). In particular, *ESR1* hotspot mutations were detected in 6/12(50%) gDNA samples extracted from CTCs isolated from CellSearch cartridges. More specifically, *ESR1* D538G hotspot mutation was detected in 6/12(50%) DNA samples (time points: 59, 61, 63, 69, 70, 74 months, Fig. 4B), the Y537S in 1/12(8%) samples (time point: 70 months, Fig. 4B), Y537C in 5/12 (42%) samples (time points: 59, 61, 63, 70, 74 months, Fig. 4B) and the Y537N in 1/12(8%) samples (time point: 61 months, Fig. 4B). All samples found positive for ESR1 mutations were collected one year after the patient started Afinitor/Aromasin therapy. At time point #28, 11 months after the first detection of *ESR1* mutations in CellSearch cartridges the patient relapsed while she was under Afinitor/Aromasin therapy. *ESR1* Y537N hotspot mutation was detected in 1/8(13%) gDNA samples extracted from EpCAM^(+)^ CTC fractions (time point: 116 months, Fig. 4B). In paired plasma-ctDNA samples, *ESR1* D538G hotspot mutation was detected in 7/9(78%) of tested cases (time points: 74, 82, 116, 118, 122, 124, 126 months, Fig. 4B).

##### *ESR1* methylation

The presence of *ESR1* methylation was investigated in 22 gDNA samples extracted from CellSearch cartridges (n = 12) and EpCAM^(+)^ cell fractions (n = 10) during the follow-up period (Fig. 4B). *ESR1* methylation was detected in 8/10 (80%) of the SB-treated gDNA samples extracted from EpCAM^(+)^ cell fractions (time points: 74, 76, 100, 103, 116, 118, 122, 126 months, Fig. 4Β). In all paired plasma-ctDNA samples (time points: 74, 76, 82, 100, 103, 116, 118, 122, 124, 126 months, Fig. 4B) *ESR1* methylation was detected with a significant agreement with the corresponding EpCAM^(+)^ cell fraction in 8/10 (80%) cases.

- Phenotypic characterization of CTCs by IF

The phenotypic characterization of CTCs using double IF staining revealed that a substantial number of CTCs were HER2^+^ even if the primary tumor was negative for HER2 amplification. In particular, we have noticed changes of HER2^+^ expression in CTCs during the follow-up period. In 2012, the patient was enrolled in a prospective randomized trial of secondary adjuvant Herceptin trastuzumab versus standard treatment in patients with HER2^(+)^ CTCs after completion of adjuvant chemotherapy^43^. At the time point of 26 months follow-up, 25CTCs/23 mL of blood were enumerated using the CellSearch (Fig. 4Α). In a paired PB sample, using the same blood draw, the phenotypic characterization of CTCs has revealed that: 1 CTC was CK^(+)^/HER2^(−)^, 13 CTCs were CK^(+)^/EGFR^(+)^, 14 CTCs were CK^(+)^/EGFR^(−)^ and 112 CTCs were CK^(+)^/M30^(−)^. No CTCs were detected for the following phenotypes: CK^(+)^/HER2^(+)^, CK^(+)^/M30^(+)^, CK^(+)^/ki67^(+)^, CK^(+)^/ki67^(−)^, CK^(+)^/VEGF^(+)^, CK^(+)^/VEGFR2^(+)^, CK^(+)^/VEGFR2^(−)^ (Fig. 3A). At the time point of 29 months (Fig. 4A), when 17CTCs/23 mL of blood were enumerated using the CellSearch, the phenotypic characterization of CTCs demonstrated that the cells were HER2^(−)^ (HER2^(−)^, EGFR^(+)^, Ki67^(−)^, M30^(−)^ and VEGFR2^(−)^) (Fig. 3A). In September 2014, after 57 months of follow-up (Fig. 4A), when 275 CTCs/7.5 mL were enumerated in peripheral blood our IF analysis has confirmed that these cells were CK^(+)^/HER2^(−)^ (Fig. 3A). Representative images of CTCs by immunofluorescence experiments and confocal laser scanning microscopy analysis are shown in Fig. 4C.

## Discussion

We present a comprehensive liquid biopsy analysis of 13 ER(+) breast cancer patients initially diagnosed with operable disease, based on the analysis of serial peripheral blood samples at different time points in a period of ten years, and correlate our findings with the clinical outcome.

In all ten patients that remained free from disease progression during the follow-up period, *CK-19* transcripts were detected only at 3/135 (2.2%) time points tested. Until now, all these patients are alive and remained free from progression disease, except Pt#8 that was lost to follow-up. On the contrary, an important increase in the copy number of *CK-19* transcripts was observed for two patients (Pt#11, Pt#13) a few years after initial diagnosis and before the confirmation of PD and diagnosis of metastasis. Our results are in agreement with previous clinical studies who have shown that the detection of *CK-19*^(+)^ mRNA cells in the peripheral blood of patients with operable breast cancer before, during and after adjuvant treatment is an independent prognostic factor associated with an increased risk of disease relapse and shorter survival^16–18,44,45^. It has also been shown that the presence of *CK-19*^(+)^ CTCs after the completion of chemotherapy is associated with increased risk of late relapse and poor survival in metastatic breast cancer^46–48^. Matikas et al. have recently shown that breast cancer patients with *CK-19*^(+)^ CTCs at baseline and at post-therapy had worse DFS and OS compared with patients with *CK-19*^(−)^ CTCs at both time-points^48^.

Our findings indicate that in 8/10 (80%) of early breast cancer patients that did not relapse no *CK-19* transcripts were detected in the EpCAM^(+)^ CTC fraction. However, there were two patients P#3 and P#6 that did not relapse during the time of the follow-up. In these two patients we detected *CK-19* transcripts in EpCAM^(+)^ CTC fractions, but in only one time point out of 14 (1/14, 7%) time points tested for P#3, and in only two timepoints out of 16 (1/16, 6%) for P#6. It is well known that most of CTCs are destroyed during circulation by immune cells, and this very low detection rate of CTC in peripheral blood could possibly be an explanation for the lack of disease recurrence in these two patients.

Three out of these thirteen patients (Pt#11, Pt#12, Pt#13) developed distant metastasis during the follow-up period. In these patients we further analyzed DNA samples extracted from EpCAM^(+)^ cell fractions and paired plasma for the detection of *PIK3CA* and *ESR1* mutations. *ESR1* mutations were not detected in any sample for Pt#11 and Pt#12. *ESR1* mutations were detected in the plasma ctDNA of Pt#13 consistently at five different time points during the follow-up period. This patient received everolimus and exemestane for a long time, and after disease progression on this therapy, *ESR1* mutations were detected in serial samples of plasma ctDNA. Our results are in accordance with those reported in the EROS1 study, where *ESR1* mutations were identified in patients previously treated with tamoxifen or aromatase inhibitors, revealing a possible correlation between long term aromatase inhibitor therapy and the existence of *ESR1* mutations^49^. Additional studies have shown that acquired *ESR1* mutations are a major mechanism of resistance to aromatase inhibitors^50–53^. Our results reflect the reports indicating that *ESR1* mutations (especially D538G, Y537S) are associated with more aggressive disease^38,53,54^. In the phase III PADA-1 trial presented at 2021 San Antonio Breast Cancer Symposium, it was observed that switching from an aromatase inhibitor plus palbociclib to fulvestrant and palbociclib upon early identification of the *ESR1* mutation in plasma-before disease progression- the median PFS was doubled. This trial has also shown that *ESR1* mutations are rarely detected in plasma-cfDNA of ER+ HER2− MBC patients with no overt resistance to aromatase inhibitor and that the detection of *ESR1* mutations was associated with a significantly shorter PFS, suggesting that the existence of *ESR1* mutation at baseline could accelerate the outset of resistance to AI-palbociclib^55^. The first results of this trial have recently been published showing that the early therapeutic targeting of *ESR1* mutations in blood results in significant clinical benefit^56^. It is an important study as the original design explored in PADA-1 might help with tackling acquired resistance with new drugs in future trials^56^. In our study, *PIK3CA* mutations were detected in all patients that later developed metastasis. More specifically in Pt#11, E545K mutation was detected in 1/10 (10%) EpCAM^(+)^ CTC fractions but not in paired plasma-ctDNA samples. In Pt#12, E545K mutation was detected in 8/14 (57%) EpCAM^(+)^ CTC fractions and in 1/14 (7%) paired plasma-ctDNA samples checked. In Pt#13, E545K mutation was detected in 2/10 plasma-ctDNA samples but not in paired EpCAM^(+)^ CTC fractions. In the same patient, H1047R mutation was detected in 3/10 (30%) EpCAM^(+)^ CTC fractions and in 2/10 (20%) paired plasma-ctDNA samples. We have already shown before in a direct comparison study using the same blood draw, and the same detection methodology that there is no a 100% concordance in the detection of *PIK3CA* mutations in CTCs and corresponding ctDNA^31^. In addition, this discordance may be explained as a result of potential intratumor heterogeneity and as an effect of therapeutic pressure on different cancerous subclones.

We further focused our analysis on Pt#13 that was diagnosed with operable BC in 2010 and died from metastatic breast cancer in 2020. CTC enumeration revealed a bad prognosis for this patient, even before the initiation of systemic chemotherapy, that was based on the relatively high number of CTCs. During the first five years (2010–2015) of follow-up, even before metastasis in the liver was histologically confirmed in September 2015 (time point: 69 months), the patient was constantly positive for the presence of CTCs, CTC counts were constantly more than 5CTCs /23 mL PB and a dramatic rise in CTC counts in September 2014 (timepoint: 57 months) has clearly suggested disease progression at least four years before imaging documentation of metastasis.

Rapid increases in CTC numbers at months 74 and 122, were associated with metastatic disease documented by biopsy 6 months earlier. In this patient, verification of recurrence and administration of systemic therapy was verified by tissue biopsy of the metastatic lesions in the liver and was not based on CTC detection. The increase of the CTC number was associated with clinically and radiologically documented disease progression and the different administered treatments were given according to the national guidelines using approved drugs and combinations.

Molecular characterization of CTCs isolated from this patient by double immunofluorescence demonstrated that practically all the detected cells were CK(+)/HER2(+) and CK(+)/Vimentin(+), while the primary tumor was HER2 negative. Based on our initial data demonstrating that trastuzumab can eliminate HER2^+^ CTCs^57^, the patient was enrolled in an open-label randomized Phase II trial of secondary adjuvant trastuzumab in early-stage breast cancer patients harboring HER2^+^ CTCs after the completion of adjuvant chemotherapy and radiotherapy^42^ and trastuzumab administration resulted in a clear decrease of the CTCs numbers lasting for 8 months. However, at the time of relapse the significant increase of CTCs was characterized by the presence of exclusively HER2- but EGFR+ CTCs. It is to note that lapatinib also resulted in decreased number of CTCs but for a short period of time. Retrospective molecular analysis of DNA derived from CellSearch cartridges, (P#13, time point: 59 months, Nov 2014) has revealed the presence of the E545K *PIK3CA* mutation in CTCs that is known to confer resistance to trastuzumab as well as *ESR1* mutations (D538G, Y537C), that are now known to confer resistance to the everolimus/exemestane combination. In September 2015 (time point: 69 months), CTC number increased again while imaging studies revealed multiple liver metastatic lesions which were histologically confirmed. CTC enumeration has indicated metastasis at least one year earlier (September 2014, time point: 57 months) than radiologically revealed and histologically confirmed (September 2015, time point: 69 months).

It is remarkable that even one year before clinical manifestation of metastasis, and till 2020, *PIK3CA* hotspot mutations (E545K and H1047R) and *ESR1* mutations (D538G, Y537C, Y537S, Y537N), were continuously detected, not only in CTC-derived DNAs from CellSearch cartridges, but also in plasma-cfDNA, and EpCAM^(+)^ CTC fractions as well. At the same time period (Feb 2016–May 2020), *ESR1* methylation, that was also shown to be associated with lack of response to hormonotherapy^41^, was detected in plasma-cfDNA and corresponding EpCAM^(+)^ CTC fractions, but not in CTC-derived DNAs from CellSearch cartridges. Based on these results, it is evident that while the rise in CTC counts indicated very early the metastatic spread, CTC molecular characterization at the DNA level clearly indicated that the tumor had already developed resistance to the treatment. During Feb 2016–May 2020 gene expression analysis in EpCAM^(+)^ CTCs fractions, has revealed the EMT nature of these cells, since the epithelial marker *CK-19* that was expressed in all samples tested, was co-expressed with stem cell markers and EMT markers. *CD24*^-/low^/*CD44*^high^ profile was detected in all samples and the *CD24*^−/low^/*ALDH1*^high^ in 50% of samples analyzed during this period of time. Our results on *CK-19, TWIST1, CD24* and *ALDH1* expression in CTCs are in accordance to those presented in a previous study^58^.

Molecular characterization of CTC at the DNA level revealed one year before the clinical manifestation of metastasis the presence of *PIK3CA* and *ESR1* mutations in CTCs that are now known to be highly important for therapy resistance. Our retrospective analysis indicates that these findings were an early indication that disease would not respond to the therapy given to this patient (Trastuzumab, lapatinib, and Afinitor/Aromacin), but at that time this was not known, and moreover Alpelisib was not FDA-approved. Alpelisib was administered to P#13 for 2 months, but severe adverse events (diabetes and septicemia necessitating patient’s hospitalization for 18 days) resulted to early treatment discontinuation. The patient died from disease progression 6 months later.

To the best of our knowledge, this is the first time that early detection of Minimal Residual Disease in breast cancer based on a comprehensive liquid biopsy analysis and a ten year follow-up of operable breast cancer patients is reported. According to our results, CTC analysis provided highly important information for the management of these patients. CTC enumeration as expected indicated at the very beginning whether these patients were at a very high risk for metastatic spread. Our results indicate that a comprehensive liquid biopsy analysis provides highly important information for the therapeutic management of breast cancer patients, and could guide oncologists to select molecular targeted treatments, based on precision medicine. Up to now, the use of CTCs to guide systemic therapy remains controversial, and there are no definitive data to support its clinical utility. However, a number of clinical trials based on liquid biopsy are ongoing and are expected to demonstrate the clinical utility of CTCs^59^. Our results indicate that comprehensive liquid biopsy analysis could reveal the presence of MRD four years before the appearance of clinically detectable metastatic disease. We strongly believe that interventional clinical trials based on a comprehensive liquid biopsy analysis are highly needed to change clinical practice for the benefit of breast cancer patients.

## Methods

### Patients

All patients were diagnosed with early and operable ER(+) breast cancer. Peripheral blood (PB) (20 mL) was obtained from all patients at different time points during a long-term follow-up period since diagnosis, two weeks after the removal of the primary tumor. Ten of these patients did not relapse and remained metastasis free during the follow-up period, while three of them relapsed within 5–7 years. In all PB samples EpCAM^(+)^ cell fractions were enriched using immunomagnetic capture beads, and analyzed for CK-19 mRNA expression, and in parallel, ctDNA, isolated from paired plasma using the same blood draw (2 mL), was analyzed for all these patients. All samples were analyzed for *ESR1* and *PIK3CA* mutations in genomic DNA (gDNA), extracted from EpCAM^(+)^ fractions and in paired plasma ctDNA at different time points. CTC enumeration using the CellSearch system (Menarini, Silicon Biosystems) was also performed. For one patient that later developed metastasis, CellSearch analysis was performed at regular intervals within a year for 10 consecutive years and phenotypic characterization of CTCs was also performed through double immunofluorescence. gDNA was extracted from the isolated CTCs from CellSearch cartridges to detect *ESR1* and *PIK3CA* mutations for this patient and all CTC-derived gDNA samples were also analyzed for *ESR1* methylation status. All patients have signed informed consent for MRD screening, and the study was conducted in accordance with the Declaration of Helsinki and has been approved the Medical Ethical Committee of the General University Hospital of Heraklion, Crete, Greece (Ethical Allowance: 8756/23-6-2014).

### Molecular analysis of CTCs

#### Gene expression

##### RNA isolation: cDNA synthesis

All steps including, the isolation of EpCAM^(+)^ fractions, total RNA extraction using Trizol-LS reagent (Invitrogen, USA) were performed as previously described^15,60,61^. cDNA synthesis was carried out with the High-Capacity RNA-to-cDNA Kit (Applied Biosystems, USA) according to the manufacturer’s protocol in 20μL of total volume reaction. All cDNA samples were kept at − 20 °C until use.

##### RT-qPCR

RT-qPCR was performed for the following genes: (a) CK-19, (b) EMT-associated marker TWIST-1, and (c) stem-cell markers CD24, CD44, ALDH-1. B2M (beta-2-microglobulin) was used as a reference gene^61^. RT-qPCR assays for the quantification of CK-19, TWIST-1, CD24, CD44 and ALDH-1 transcripts were performed as previously described^14,15,62^. The cut-off for CD24, CD44 and ALDH-1 was estimated in respect to HPRT expression as previously reported^61^ in a group of 10 healthy individuals whose peripheral blood has been analyzed in exactly the same way as patient’s.

#### DNA analysis

##### DNA isolation

Genomic DNA (gDNA) was isolated from CellSearch cartridges, and EpCAM^(+)^ CTC fractions at regular timer intervals from initial diagnosis during the follow-up period as follows: (a) CellSearch cartridges: The pre-enriched sample (pre-stained CTCs and WBCs) was aspirated from the corresponding CellSearch cartridges, gDNA was extracted using the QIAamp DNA Micro Kit (Qiagen, Germany) in accordance with manufacturer’s instructions^42^. (b) EpCAM^(+)^ fractions: Similarly, gDNA from all available EpCAM^(+)^ fractions was extracted from CTCs in Trizol LS (Invitrogen, USA) as previously described^30,63^. In parallel to the isolation of CTC-derived gDNA, plasma cell-free DNA was isolated as previously described^31^. The QIAamp Circulating Nucleic Acid Kit (Qiagen) was used to isolate ctDNA from 2.0 mL of plasma according to the manufacturer’s instructions. DNA concentration was measured in all samples, using the Nanodrop ND-1000 spectrophotometer (Thermo Scientific, USA) and DNA integrity was assessed prior to the analysis by amplifying a wild-type region in exon 20 of PIK3CA gene^64^.

##### *PIK3CA* and *ESR1* mutations

All available DNA samples were analyzed for *PIK3CA* hotspot mutations (c.1633G > A: E545K exon 9 and c.3140A > G: H1047R exon 20) as well as for *ESR1* hotspot mutations [Y537S (c.1610A > C), Y537C (c.1610A > G), Y537N (c.1609T > A) and D538G (c.1613A > G)] as previously described^30,33^. For plasma ctDNA analysis, a drop-off ddPCR for screening the *ESR1* mutations in exon 8 (Y537S, Y537C, Y537N, D538G and L536R) was performed as previously reported^65^ in order to detect the presence of the *ESR1* mutations before identifying individual mutations using the ESR1-NAPA assay^33^.

##### *ESR1* methylation

All available DNA samples were also processed to sodium bisulfite (SB) treatment using the EZ DNA Methylation Gold Kit (ZYMO Research Corp., USA) according to manufacturer’s instructions. Only samples that were positive for exon 20 *PIK3CA* amplification were further processed to SB-treatment. SB-treated DNA was stored at − 80 °C until further use. After the SB-treatment, SB-converted DNA integrity was assessed by a real-time methylation-specific PCR (MSP) for b-actin (ACTB) as previously described^41,60^. Subsequently, all SB-treated samples were analyzed for ESR1 promoter methylation using real-Time MSP, based on our previously developed and validated protocols^41,66^.

### CTC enumeration

CTC enumeration was performed using the FDA-cleared CellSearch system (Menarini, Silicon Biosystems) in PB samples collected during all these years at regular time intervals for most patients. For Pt#13 CellSearch analysis was performed for 10 consecutive years in peripheral blood draws in sequential patient samples During the first 5 years of follow-up, before the development of clinically confirmed metastatic disease, 23 ml of peripheral blood collected in CellSave preservative tubes was analyzed. Conversely, during the metastatic phase of Pt#13 disease 7.5 mL of peripheral blood were used for CTC enumeration every 3–6 months.

### Double immunofluorescence (IF)

Phenotypic characterization of CTCs was performed only for Pt#13. CTCs isolated from 20 ml of peripheral blood were used for their phenotypic characterization during the follow-up. The first 5 mL of blood were discarded to avoid contamination with epithelial cells from the skin. Peripheral blood mononuclear cells (PBMCs) were isolated with Ficoll-Hypaque density gradient (d = 1.077 g/mL) centrifugation at 1800 rpm (600 g) for 30 min. Cytospins of 5 105 PBMCs were prepared and stored at − 80 °C until their use for double staining experiments. The presence of CK-positive cells in PBMCs’ cytospins was investigated using the A45-B/B3 mouse antibody (anti-CK-8, CK-18, CK-19, Micromet Munich) and is further referred as CK+ in the text. At some time points the presence of CTCs in PBMCs’ cytospins was investigated using monoclonal antibodies against ki67 (proliferation marker, Abcam) or/and M30-FITC conjugated (apoptotic marker, Roche Diagnostics, Basel). Double immunofluorescence staining was performed as previously described^67–72^.

## Supplementary Information

Supplementary Information.

## Data Availability

The data presented in this study are available on request from the corresponding author. The data are not publicly available due to ethical restrictions.
