# Supplementary material for: Comprehensive liquid biopsy analysis as a tool for the early detection of minimal residual disease in breast cancer
Source: Sci Rep. 2023 Jan 23;13:1258. doi: 10.1038/s41598-022-25400-1 (PMC9870904; doi:10.1038/s41598-022-25400-1)
Supplement: Supplementary file 1 — Supplementary Information. [file 41598_2022_25400_MOESM1_ESM.docx]

**Comprehensive liquid biopsy analysis as a tool for the early detection of Minimal Residual Disease in breast cancer**

Dimitra Stergiopoulou^1^, Athina Markou^1^, Areti Strati^1^, Martha Zavridou^1^, Eleni Tzanikou^1^, Sophia Mastoraki^1^, Galatea Kallergi^2^, Vassilis Georgoulias^3^, and Evi Lianidou^1*^

^1^ Analysis of Circulating Tumor Cells Lab, Department of Chemistry, National and Kapodistrian University of Athens, 15771, Greece

^2^ Division of Genetics, Cell and Developmental Biology, Department of Biology, University of Patras, Patras, 26500, Greece

^3^ First Department of Medical Oncology, METROPOLITAN General Hospital, 264, Mesogion Av, Cholargos, Athens, Greece.

**Correspondence to:**

**Evi Lianidou, PhD,**

**Analysis of Circulating Tumor Cells Lab, Lab of Analytical Chemistry,**

**Department of Chemistry, National and Kapodistrian University of Athens,**

**15771, Athens, Greece Email:** [**lianidou@chem.uoa.gr**](mailto:lianidou@chem.uoa.gr)

**Supplementary Table S1** P#13: CTC enumeration and molecular characterization during the follow-up and respective therapeutic strategies. Relapses are highlighted in bold.

| Sampling  Time point | **Month**  **/ Year** | **follow-up,**  **months** | **CTCs enumeration**  **(CellSearch)** | **Molecular characterization** | **Therapy** |
| --- | --- | --- | --- | --- | --- |
| 1 | 01/2010 | 0 | 10 | CTC phenotypic analysis, IF | Chemotherapy, FEC/TXT |
| 2 | 03/2011 | 14 | 22 | CTC phenotypic analysis, IF  CK^(+)^/HER2^(+)^ and CK^(+)^/Vimentin^(+)^ | trastuzumab |
| 3 | 04/2011 | 15 | 21 | CTC phenotypic analysis, IF | trastuzumab |
| 4 | 07/2011 | 18 | 4 | CTC phenotypic analysis, IF  40 CTCs CK^(+)^/HER2^(+)^, 2 CTCs HER2^(+)^ | trastuzumab |
| 5 | 08/2011 | 19 | 19 | CTC phenotypic analysis, IF | trastuzumab |
| 6 | 09/2011 | 20 | 30 | CTC phenotypic analysis, IF  0 CTCs CK^(+)^/HER2^(+)^ | trastuzumab |
| 7 | 10/2011 | 22 | 34 | CTC phenotypic analysis, IF  1 CTC CK^(+)^ | trastuzumab |
| 8 | 12/2011 | 23 | 11 | CTC phenotypic analysis, IF  1 CTC CK^(+)^/HER2^(+)^ | trastuzumab |
| 9 | 01/2012 | 24 | 14 | CTC phenotypic analysis, IF  0 CTCs CK^(+)^/HER2^(+)^, 1 CTC EGFR^(-)^ | trastuzumab |
| 10 | 02/2012 | 25 | 15 | CTC phenotypic analysis, IF  0 CTCs CK^(+)^/HER2^(+)^ | lapatinib |
| 11 | 02/2012 | 25 | 25 | CTC phenotypic analysis, IF  1 CTC CK^(+)^/HER2^(-),^ 13 CTCs CK^(+)^/EGFR^(+)^, 14 CTCs CK^(+)^/EGFR^(-)^ and 112 CK^(+)^/M30^(-)^ | lapatinib |
| 12 | 04/2012 | 28 | 28 | CTC phenotypic analysis, IF | lapatinib |
| 13 | 06/2012 | 29 | 17 | CTC phenotypic analysis, IF  HER2^(-)^, EGFR^(+)^, Ki67^(-)^, M30^(-)^ and VEGFR2(-) | lapatinib |
| 14 | 06/2012 | 30 | 22 | CTC phenotypic analysis, IF  2 CTCs CK^(+)^/HER2^(+)^ | Afinitor/  Aromasin |
| 15 | 07/2012 | 31 | 14 | CTC phenotypic analysis, IF | Afinitor/  Aromasin |
| 16 | 09/2012 | 33 | 16 | CTC phenotypic analysis, IF | Afinitor/  Aromasin |
| 17 | 11/2012 | 34 | 9 | CTC phenotypic analysis, IF | Afinitor/  Aromasin |
| 18 | 01/2013 | 37 | 8 | CTC phenotypic analysis, IF | Afinitor/  Aromasin |
| 19 | 04/2013 | 40 | 0 | CTC phenotypic analysis, IF | Afinitor/  Aromasin |
| 20 | 09/2013 | 45 | 5 | CTC phenotypic analysis, IF | Afinitor/  Aromasin |
| 21 | 01/2014 | 49 | 21 | CTC phenotypic analysis, IF | Afinitor/  Aromasin |
| 22 | 05/2014 | 52 | 17 | CTC phenotypic analysis, IF | Afinitor/  Aromasin |
| 23 | 09/2014 | 57 | 275 | CTC phenotypic analysis, IF  11 CTCs CK^(+)^/HER2^(-)^ | Afinitor/  Aromasin |
| 24 | 11/2014 | 59 | 244 | CTC phenotypic analysis, IF  2 CTCs CK^(+)^/HER2^(+)^ | Afinitor/  Aromasin |
| 25 | 01/2015 | 61 | 221 | DNA mutations^b^  DNA methylation^c^  CTC phenotypic analysis, IF  1 CTC CK^(+)^/HER2^(+)^ | Afinitor/  Aromasin |
| 26 | 04/2015 | 63 | 127 | DNA mutations^b^  DNA methylation^c^ | Afinitor/  Aromasin |
| 27 | 06/2015 | 66 | 157 | DNA mutations^b^  DNA methylation^c^ | Afinitor/  Aromasin |
| 28 | **09/2015** | 69 | 250 | DNA mutations^b^  DNA methylation^c^ | Afinitor/  Aromasin  **(PD, Liver Metastasis)** |
| 29 | 10/2015 | 70 | 148 | DNA mutations^b^  DNA methylation^c^ | Taxol weakly Avastin |
| 30 | **02/2016** | 74 | 1,254 | Gene expression^a^  DNA mutations^b^  DNA methylation^c^ | Chemotherapy (Taxol weakly Avastin) |
| 31 | 04/2016 | 76 | 411 | Gene expression^a^  DNA mutations^b^  DNA methylation^c^ | Taxol/Avastin |
| 32 | 09/2016 | 82 | 108 | Gene expression^a^  DNA mutations^b^  DNA methylation^c^ | Taxol/Avastin (Response plateau) |
| 33 | 03/2018 | 100 | 100 | Gene expression^a^  DNA mutations^b^  DNA methylation^c^ | Xeloda/Avastin |
| 34 | 07/2018 | 103 | 82 | Gene expression^a^  DNA mutations^b^  DNA methylation^c^ | Xeloda/Avastin |
| 35 | 11/ 2018 | 108 | 165 | Gene expression^a^  DNA mutations^b^  DNA methylation^c^ | Xeloda/Avastin |
| 36 | 07/2019 | 116 | 478 | Gene expression^a^  DNA mutations^b^  DNA methylation^c^ | Xeloda-Avastin |
| 37 | **09/2019** | 118 | 825 | Gene expression^a^  DNA mutations^b^  DNA methylation^c^ | NO THERAPY |
| 38 | **01/2020** | 122 | 1,423 | Gene expression^a^  DNA mutations^b^  DNA methylation^c^ | Imbance/Faslodex |
| 39 | 03/2020 | 124 | 703 | Gene expression^a^  DNA mutations^b^  DNA methylation^c^ | Alpelisib |
| 40 | **05/2020** | 126 | 800 | Gene expression^a^  DNA mutations^b^  DNA methylation^c^ | No therapy |
| 41 | 09/2020 | 130 | Not performed | Not performed | Death |

a: performed in EpCAM+ CTC fractions

b: performed in DNA isolated from CellSearch cartridges, EpCAM+ CTC fractions, and plasma cell-free DNA

c: performed in SB- converted DNA isolated from CellSearch cartridges, EpCAM+ CTC fractions, and plasma cell-free DNA
